# Sense of Agency and Its Disturbances: A Systematic Review Targeting the Intentional Binding Effect in Neuropsychiatric Disorders.

**DOI:** 10.1192/j.eurpsy.2024.1290

**Published:** 2024-08-27

**Authors:** M. Di Luzio, L. Moccia, E. Conte, M. Modica, M. Ambrosecchia, M. Ardizzi, P. Lanzotti, G. Kotzalidis, D. Janiri, M. Di Nicola, L. Janiri, G. Sani, V. Gallese

**Affiliations:** ^1^Child and Adolescent Neuropsychiatry Unit, Bambino Gesù Children’s Hospital, IRCCS; ^2^Department of Neuroscience, Section of Psychiatry, Università Cattolica del Sacro Cuore; ^3^Department of Psychiatry, Fondazione Policlinico Universitario Agostino Gemelli IRCCS; ^4^NESMOS Department, University of Rome La Sapienza, Faculty of Medicine and Psychology, Sant’Andrea University Hospital, Rome; ^5^Department of Medicine and Surgery, Unit of Neuroscience, University of Parma, Parma, Italy; ^6^Italian Academy for Advanced Studies in America at Columbia University, New York, United States

## Abstract

**Introduction:**

The sense of agency (SoA) indicates a person’s ability to feel her/his own motor acts as actually being her/his, and through them to exert control over the course of external events. Disruptions in SoA may profoundly affect the individual’s functioning, as observed in several neuropsychiatric disorders.

**Objectives:**

This is the first article to systematically review studies that investigated intentional binding (IB), a quantitative proxy for SoA measurement, in neurological and psychiatric patients.

**Methods:**

Eligible were studies of IB involving patients with neurological and/or psychiatric disorders. The research adhered to the guidelines outlined in the Preferred Reporting Items for Systematic Reviews and Meta-Analyses (PRISMA).

**Results:**

We included 15 studies involving 692 individuals. Risk of bias was low throughout studies. Eligible studies dealt with data from 357 patients with neuropsychiatric disorders matched with 335 HCs. Of included patients, 95 were with schizophrenia (SCZ), 30 with a putative prodromal psychosis (PP), 21 with borderline personality disorder (BPD), 66 with Parkinson’s disease (PD), 38 with an autism spectrum disorder (ASD), 29 with functional movement disorders (FMDs), 25 with Gilles de la Tourette syndrome (GTS), 52 with anorexia nervosa (AN; 22 with active disorder and 30 after they had recovered), and 10 with Cortico-Basal syndrome (CBS).

Temporal binding was calculated in eleven studies using variations of the experimental procedure introduced by Haggard et al. (Haggard et al. *Nat Neurosci* 2002;5 382-385)(Figure 1, A), while four studies utilized a different paradigm named interval estimation (IE)(Figure 1, B).

**Image:**

**Figure:**
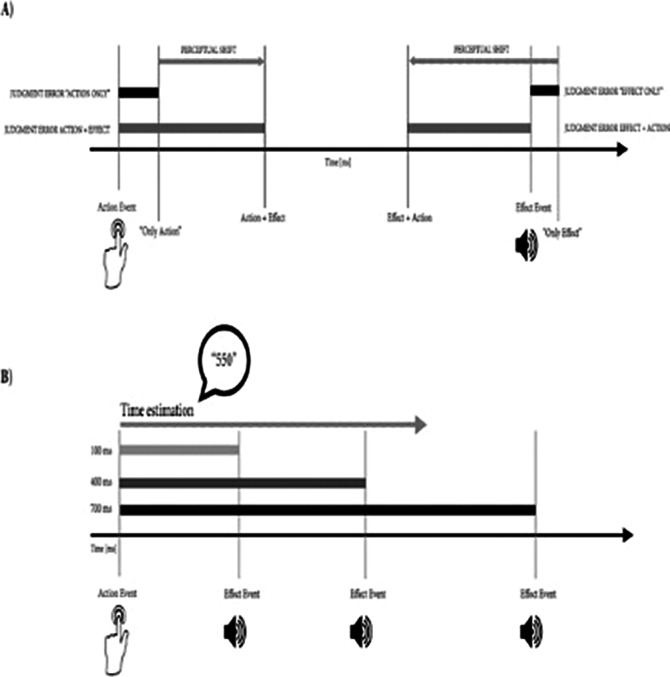

**Conclusions:**

Abnormally increased action-outcome binding was found in schizophrenia and in patients with Parkinson’s disease taking dopaminergic medications or reporting impulsive-compulsive behaviours. A decreased IB effect was observed in Tourette’s disorder and functional movement disorders whereas increased action-outcome binding was found in patients with cortico-basal syndrome. The extent of IB deviation from healthy control values correlated with the severity of symptoms in several disorders. Inconsistent effects were found for autism spectrum disorders, anorexia nervosa, and borderline personality disorder. Findings pave the way for treatments specifically targeting SoA in neuropsychiatric disorders where IB is altered.

**Disclosure of Interest:**

None Declared

